# The Dynamic Capsid Structures of the Noroviruses

**DOI:** 10.3390/v11030235

**Published:** 2019-03-08

**Authors:** Hong Q. Smith, Thomas J. Smith

**Affiliations:** Department of Biochemistry and Molecular Biology, University of Texas Medical Branch at Galveston, 301 University Boulevard, Galveston, TX 77555-0645, USA; hqsmith@UTMB.EDU

**Keywords:** caliciviruses, antibody neutralization, dynamics, viral receptors

## Abstract

Noroviruses are responsible for almost a fifth of all cases of gastroenteritis worldwide. New strains evolve every 2–4 years by escaping herd immunity and cause worldwide epidemics. In the US alone, noroviruses are responsible for ~20 million cases and more than 70,000 hospitalizations of infected children, annually. Efforts towards a vaccine have been hindered by a lack of detailed structural information about antibody binding and the mechanisms of antibody escape. Caliciviruses have 180 copies of the major capsid protein (VP1; ~58 kDa), that is divided into the N-terminus (N), the shell (S) and C-terminal protruding (P) domains. The S domain forms a shell around the viral RNA genome, while the P domains dimerize to form protrusions on the capsid surface. The P domain is subdivided into P1 and P2 subdomains, with the latter containing the binding sites for cellular receptors and neutralizing antibodies. There is increasing evidence that these viruses are extremely dynamic and this flexibility is critical for viral replication. There are at least two modes of flexibility; the entire P domain relative to the shell and within the P domain itself. Here, the details and possible roles for this remarkable flexibility will be reviewed.

## 1. Introduction

Noroviruses are the major cause of epidemic gastroenteritis in humans and, as such, are important pathogens (for review, see [1]), causing ~20 million cases annually, resulting in more than 70,000 hospitalizations and 570–800 deaths in the US alone. While not often a fatal disease in the developed world, norovirus infections are estimated to cost more than $2 billion per year for healthcare and lost productivity. Controlling the spread of norovirus is challenging since as few as ten virions are sufficient to infect an adult [2].

Efforts to make effective norovirus vaccines have been thwarted by our lack of understanding of the structural mechanisms of viral escape. In addition, noroviruses are constantly evolving and generate new strains every 2–4 years [3,4,5] that result in worldwide epidemics [5,6]. Developing efficacious vaccines requires a detailed understanding of how escape mutations block antibody binding and the limitations in altering the virus capsid to evade the immune system. Such studies have been difficult with human noroviruses. While there have been advances in cell culture methods [7,8], the lack of small animal models have made *in-vivo* analyses more difficult [9]. Nevertheless, there has been a great deal of progress with vaccine development using virus like particles. For a review see [10].

**Figure 1:**
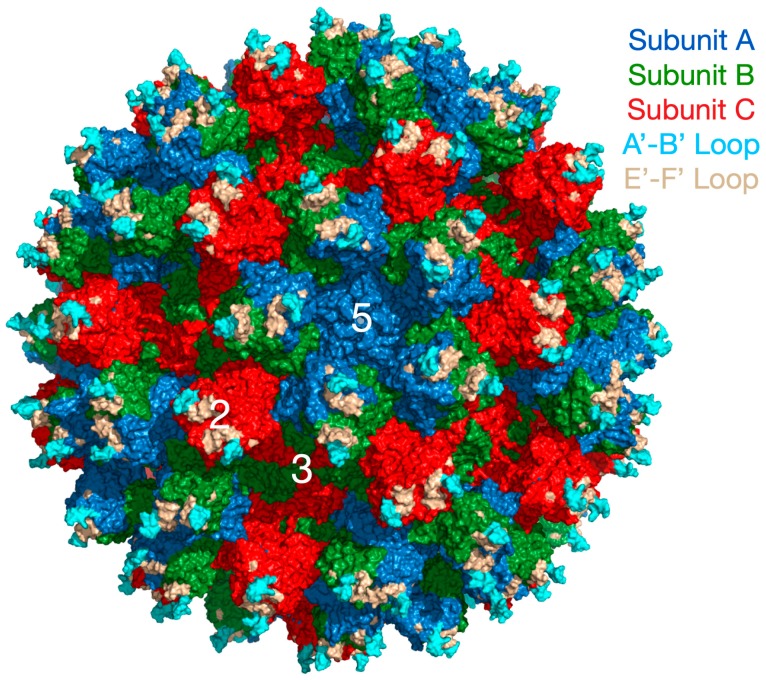
Overall architecture of the Calciviruses. This figure shows the entire capsid of mouse norovirus (MNV) based on the cryo-EM structure (PDB:6CRJ, [14,20]). The subunits A, B, and C are shown in blue, green, and red, respectively. The P domain dimers are composed of A and B around the 5-fold axes and of C dimers at the 2-fold axes. Also highlighted are the A’–B’ (cyan) and E’–F’ (tan) loops discussed in the text.

Caliciviruses are T = 3 icosahedral particles with 180 copies of the major capsid protein (VP1; ~58 kDa), that is divided into the N-terminus (N), the shell (S) and C-terminal protruding (P) domains [11,12,13,14]. The S domain forms a shell around the viral RNA genome, while the P domains dimerize to form protrusions on the capsid surface. The P domain is subdivided into P1 and P2 subdomains, with the latter containing the binding sites for cellular receptors [15,16] and neutralizing antibodies [17,18,19]. The overall architecture of mouse norovirus is shown in Figure 1 with the three copies of VP1 in the icosahedral asymmetric unit being designated as subunits A (blue), B (green), and C (red). Also noted in this figure is the location of the A’–B’ and E’–F’ loops in the P2 domain that will be discussed in detail below.

The purpose of this review is to examine recent results demonstrating that the Calicivirus capsid is a dynamic structure and that this flexibility may play important roles in receptor binding and escape from immune surveillance. From these studies, there are at least two aspects of capsid flexibility; the entire P domain freely moves about the capsid surface and the conformation of the P domain itself is highly flexible and sensitive to antibody escape mutations and receptor binding.

## 2. The First Mode of Flexibility; “Floating” P Domains

**Figure 2:**
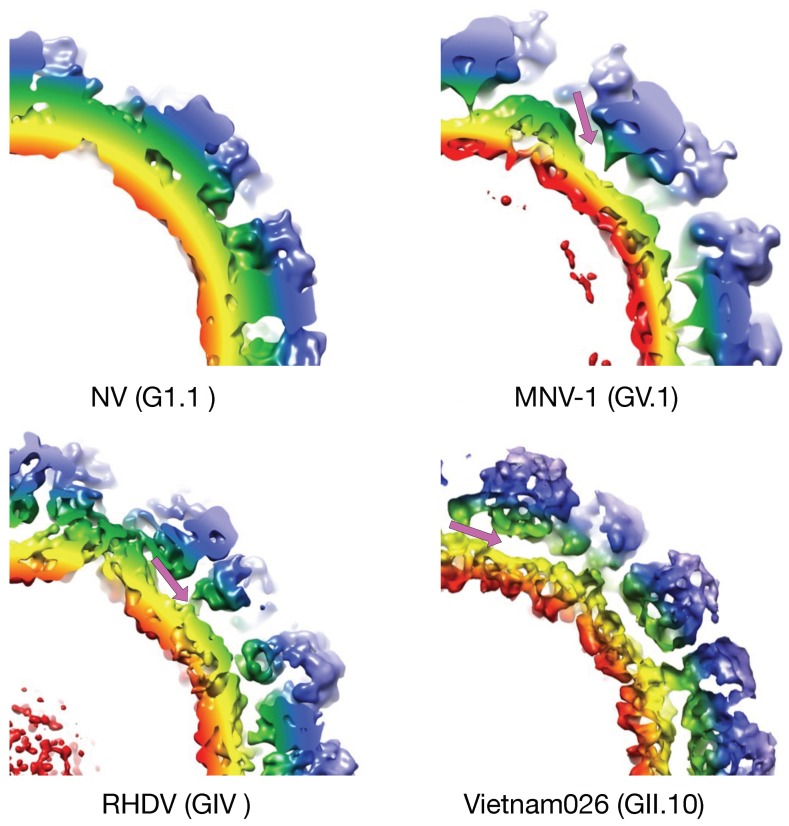
The P domain is observed to be “floating” above the shell surface in several different genotypes. Shown here are the cryo-EM image reconstructions of MNV, RHDV, and human Vietnam026 and of the crystal structure of NV. The gap between the P domains and the shell in these viruses are noted by the mauve arrows. The surface of NV is calculated from the atomic structure and all of the images are colored according the distance from the center of the particle. Note that while the P domain of NV is not detached from the shell, it contains the flexible linker between the P domain and the shell as in the other genotypes.

**Figure 3:**
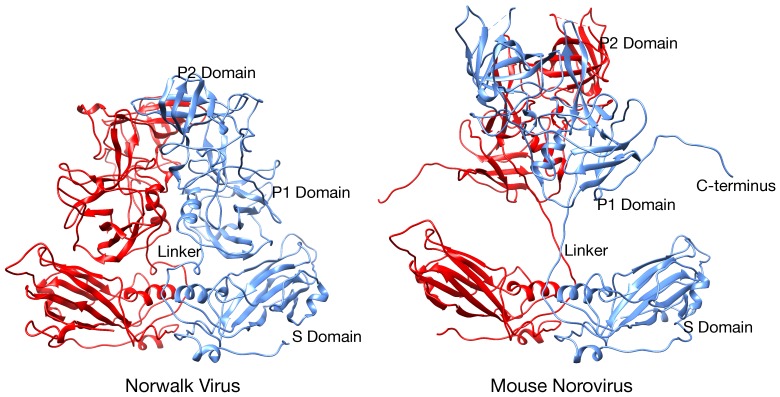
Comparison between the compressed and expanded states. Shown in this figure is the crystallographic structure of NV [11] and the pseudo atomic model of MNV based on the cryo-EM structure [14,19,20] to demonstrate the extreme movement in the P domain off the shell surface in MNV. The A and B subunits are colored red and blue to show the dimer interactions.

MNV-1 is an important norovirus model system since it can be propagated in a cell culture system, aspects of its pathogenesis and the host immune response can be examined in an animal model, large amounts of virus can be readily produced, neutralizing monoclonal antibodies have been isolated, and an infectious clone has been developed [21]. Therefore, in spite of the fact that structures of a number of other members of the *Caliciviridae* family had been determined (e.g. Norwalk virus, NV [11], and San Miguel sea lion virus, SMSV [22]), it was necessary to determine the structure of MNV-1 for comparison. Surprisingly, even from the initial examination of the MNV-1 electron density (Figure 2), it was quite apparent that the structure of MNV-1 was significantly different than the NV virus like particle (VLP) crystal structure [14,20]. While the P domains of NV VLPs rest upon the shell domain, there was a large gap in the electron density between the shell and protruding domains of MNV-1 (see mauve arrows in Figure 2). When the NV VLP structure was overlaid onto the MNV-1 density, it was clear that the entire P1 domain lies in that gap between the P and S domains and that the electron density for the MNV-1 protruding domains extended far beyond the outer extents of the P2 domains. This gives the appearance that the MNV-1 P domains lift up off the shell (S domains) to form a second proteinacous layer. The two conformations represented by NV (compressed) and MNV (expanded) is further exemplified by the atomic models of the two viruses (Figure 3). As shown in this figure, there is a linker region between the S and P domains in both viruses but is coiled up in NV and extended in MNV.

Since this unusual “floating P domain” conformation (“expanded state”) was so different than the crystal structure of NV (“compressed state”), the structure of rabbit hemorrhagic disease virus (RHDV) VLP was also determined [20]. The structure of RHDV is somewhat between what was observed for MNV-1 and NV. Similar to MNV-1, the P domains are lifted off the surface of the shell, but similar to NV, the P domains are not rotated. This orientation places the bottom edge of the A subunit P1 domain in close proximity to the S domain near the 5-fold axes, causing false connectivity at lower contouring. In addition, the P domain dimers of NV and RHDV have a more arch like shape than MNV-1. Similar to MNV-1, the electron density of the shell is defined, while the density of the P domains is diffuse, suggesting a high degree of flexibility. Unlike MNV-1, the electron density of the C/C dimers in RHDV are far more diffuse than that observed with the A/B dimers. It is notable that, unlike the RHDV A/B dimers, the RHDV C/C dimers do not make a close contact between the P1 domains and the S domain. It perhaps because of this, that the C/C dimers are apparently more mobile than the A/B dimers, and hence the diffuse density. Unlike MNV-1, the connector between the S and P1 domains is not clear. There is a slight bump in the S domain electron density where the connector should be extending from the shell and there are some islands of density in the appropriate region at lower contouring. Nevertheless, it is clear that this genotype is more like MNV with highly flexible P domains that do not contact the shell surface.

The structures of MNV (genotype GV.1) and RHDV (genotype GIV) have this “extended” conformation and represent two different genotypes. However, neither infect humans and it was possible that it is a feature not found in the human noroviruses. It was therefore important that the cryo-EM structure of the human Vietnam026 (GII.10) VLP was determined [23]. The cryo-EM reconstruction of the GII.10 norovirus VLP at ~10 Å resolution showed a number of features very similar to MNV and RHDV. The GII.10 S domain was noticeably surface exposed at the three- and five-fold axes. As with MNV and RHDV, the P domain appeared as a second outer shell, and a half-section through the VLP revealed that the P domain was raised off the S domain by ~15 Å. Importantly, these studies also showed that this apparent P domain flexibility may play an important role in antibody binding. They determined the structure of the P domain complexed with the Fab fragment from an antibody (5B18) that broadly recognized a number of different GII viruses. The crystal structure of the P domain/Fab complex [23] showed that the 5B18 Fab bound to a conserved region of the protruding domain. This binding site is involved in interactions with other regions of the capsid and partially buried in the virus particle in the “compressed” state where the P domain sits on top of the shell. Despite the occluded nature of the recognized epitope in the VLP structure, ELISA binding indicated that the 5B18 antibody was able to capture intact VLPs. The base of the P domain is only exposed to the antibody if there is extreme flexibility in the tether region between the shell and the P domain. This result has been further substantiated by more recent results in genotypes GI.1 [24] and GII.4 [25] where various epitopes are clearly buried in the compressed particle and only exposed with if the P domain is allowed to lift off the shell.

**Figure 4:**
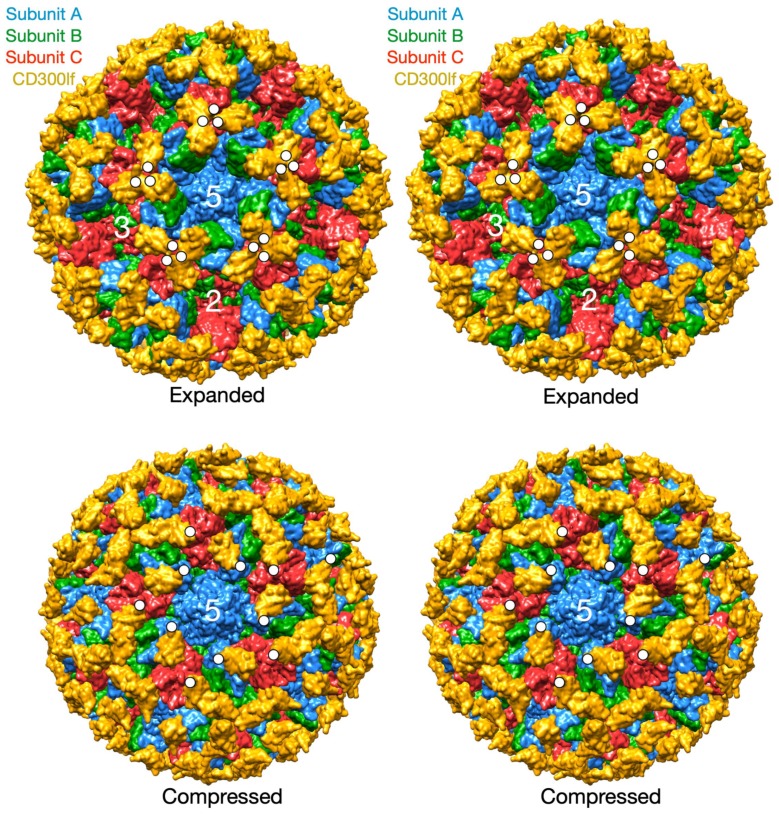
Models of the bound receptor in the compressed and expanded virions. The structures of the MNV/receptor complex in the expanded state using the cryo-EM structure and the compressed state using the relative P domain orientation observed in NV. The A, B, and C subunits are blue, green, and red respectively and the receptor is shown in yellow. The C-termini of several copies of CD300lf are highlighted by white circles.

There is also recent evidence that this flexibility of the P domain likely plays a critical role in receptor interaction in MNV [26]. In this study, the atomic structure of the isolated MNV P domain/CD300lf receptor was modeled into the pseudo-atomic cryo-EM structure of the intact MNV-1 capsid. CD300lf is a member of the immunoglobin superfamily and is found on myeloid cells. This cell membrane protein has an extracellular domain of ~170 amino acids of which ~100 residues are represented in Figure 4. The C-termini of the CD300lf N-terminal fragment is noted as white circles on some of the copies of the receptor on the viral surface. As shown in the model of the expanded form, the C-termini of the CD300lf form trimers in the icosahedron. There is clearly not enough room for all of the P domains to be saturated with receptor. This In contrast, using the position of the P domain in NV as a guide, there is a great deal of space around each of the P domains for at least a higher degree of saturation with receptor. Interestingly, the P domains around the A subunits around the 5-fold axes are oriented such that there is clearly room for saturation. While there are currently no published reports that the Caliciviruses exist in both compressed and expanded conformations, the conservation of the flexible linker, the multiple reports of antibodies recognizing buried epitopes, and unpublished results (Sherman and Smith) all point to the existance of such a transition in all Caliciviruses. Finally, this figure shows just two conformations but it is more than likely that the flexibility of the linker allows for a wide array of conformations between these two extremes. This is likely why the P domains have extremely weak density in all of the genotypes shown in Figure 2.

**Figure 5:**
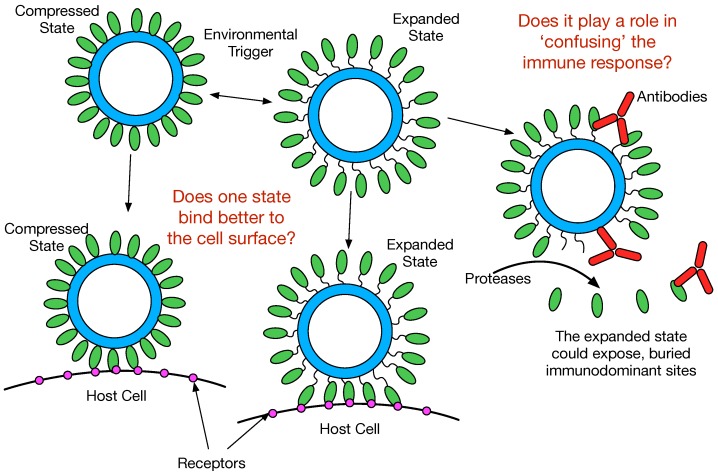
Possible roles for the conformational flexibility of the P domains with respect to the shell. The biological role for the compressed/expanded state transition is unknown. This figure shows some possibilities. In the expanded state, the P domain lifts off the shell by at least 16Å and rotates by nearly 90°. This change in orientation and flexibility might favor cell surface binding of one state versus the other. This marked flexibility might expose antigenic sites observed in the expanded state but not the compressed state [23], making some antibodies non-neutralizing [27]. The flexibility of the linker may make it more susceptible to proteolysis, thereby creating a “smoke screen” or exposure of non-neutralizing epitopes.

The conservation of this unique flexibility of the P domain across *Calicivirus* genera, antibody recognition of buried epitopes, and receptor interactions all strongly suggest that P domain flexibility plays some critical role in the *Calicivirus* life cycle. While studies are underway to ascertain this function, Figure 5 summarizes a few of the possibilities. It seems highly likely that the *Caliciviruses* can adopt both conformations depending upon environmental cues. As suggested in this figure, the viruses might prefer the compressed or expanded state when binding to the cell surface. In this way, the virus may become primed for cell attachment when in the appropriate location in the gut. From the recent MNV P domain/ CD300lf structure [26], it appears that CD300lf binding does not favor the expanded conformation and the flexibility of the P domain helps to optimize receptor binding. It should be noted that when the P domains rise off the shell, they also rotate by nearly 90°. Therefore, not only are there differences in flexibility between these two states, but also in the relative orientations of the various binding sites on the P domain.

In the expanded state, several events might occur. The flexible tether between the shell and the P domains could be sensitive to proteases. This could release soluble P domains that might act as a “smoke screen” for the immune system and present antigenic sites not normally exposed on the viral surface. Similarly, the marked flexibility of the P domains in the expanded state might expose these antigenic sites without having to be cleaved from the shell. In either case, if any of these normally buried sites are immunodominant, then the immune response might focus on producing antibodies that bind poorly or not at all to the compressed capsid. This hypothesis is supported by the findings that the immune response to MNV included antibodies to the buried shell domain [27] and from studies on human genotypes GII.10 [23], GI.1 [24], and GII.4 [25] where antibodies were found to bind to portions of the P domain that are buried in the compressed conformation. In essence, the highly flexible nature of the P domain could be a moving target for the immune response.

## 3. The Second Mode of Flexibility; within the P Domains

From the results above, the P domain is like a balloon floating above the viral shell, attached only by a thin tether. The P domain is structurally isolated from the rest of the capsid and therefore it seems highly unlikely that antibodies or receptors can bind to the P domain and transmit conformational changes to the shell. However, there is growing evidence that the P domain itself is highly flexible and that this motility is necessary for viral function.

To detail the possible roles of this flexibility within the P domain, it is important to first review the locations of antibody and receptor binding sites on the P domain. There has been a great deal of work describing virus interactions with two types of cellular receptors; complex polysaccharides and cell surface proteins.

## 4. Interaction between Human Noroviruses and Polysaccharides

As will be discussed below, there are very recent results that show that flexibility within the P domain may help human noroviruses bind to the histo-blood group antigens (HBGA) [28]. Susceptibility of an individual to NV infection is related to the expression of particular carbohydrates [29]. NV VLPs bind to the H, Lewis, and A HBGAs; the variation in which is determined by the individual’s expression of various glycotransferase enzymes (for a review, see [1]). The highest levels of expression on the small intestinal villi lie at the outermost tip. Mutagenesis studies using the structural model for NV has further articulated the virus/carbohydrate interactions [30]. These researchers have proposed that there is a pocket in the P2 domain that contains an RGD/K motif that binds the histo-blood group antigens. Other noroviruses display different ABH and Lewis carbohydrate specificities [1,31,32]. These results suggest that natural resistance and the occasional lack of correlation between an antibody response and protection is likely due to genetically determined expression of particular carbohydrate moieties [29]. Therefore, the importance of blood group carbohydrates in infection with human noroviruses is clear but whether these carbohydrates represent a viral receptor, or are sufficient for infection is not clear since there is no cell culture system for human noroviruses. It should be noted that a very small proportion (4–6%) of specifically bound VLPs is internalized in various cells [33]. While this interaction may be sufficient for infection, it is lower than the level of internalization of positive control viruses such as rotaviruses.

There have been a number of structures of norovirus P domains complexed with simple saccharides [15,34,35]. Cloned, expressed P domains, separate from the full-length capsid protein, have been crystallized and diffract to high resolution and therefore more amenable for detailed studies of oligosaccharide—P domain interactions. Norwalk virus, the archetype genogroup I norovirus, recognizes A-type and H-type human blood group antigens while VA387, a genogroup II norovirus, recognizes a larger array of blood group antigens. In the first structure [34], the P domains from VA387 were crystallized in the presence of trisaccharides representing blood group antigens A and B. These glycans bind to essentially the same location; at the interface between the two subunits and at outer most tip of the P2 domain. The both types of glycans are able to bind likely due to the fact that most of the protein/ligand interactions are via the common fucose moiety. While the binding pocket lies at the P2 dimeric interface, the predominant interactions lie on one side of the dimer. Similar studies were performed on Norwalk P domains [35]. One of the major differences between NV and VA387 is that VA387 can bind to both A and B blood group antigens while NV can only bind to A. This is well explained by the structure that shows that, while blood group antigen A binds to NV in approximately the same location as was observed in VA387, the majority of the interactions are formed between the α-GalNAc moiety and the P2 domain—the defining difference between the A and B blood group antigens. In a subsequent study [15] they determined the structure of both A and H blood group antigens complexed with NV P domains. The H blood group polysaccaride has a terminal α-fucose sugar being followed by a β-galactose. These two sugars together replaced the interactions made by the single α-GalNAc moiety in the A group antigen. Both glycans interact with a conserved tryptophan (375) and common hydrogen binding patterns with both glycans. It is therefore a very interesting case of where there is ligand specificity yet two quite different ligands bind to the same site. Similarly, the primary interactions between the HGBAs and GII.10 and GII.12 genotypes are with the terminal αfucose1-2 moiety of the HGBA at the dimer interface [36]. The remaining interactions with other HGBAs are variable with fewer hydrogen bonds.

## 5. Feline Calicivirus Interactions with Its Receptor

Feline calicivirus (FCV) causes respiratory illness and stomatitis in cats [37]. FCV interacts with a 2,6 sialic acid [38]. These glycan interactions are thought to be important but unlikely to represent the sole cellular receptor for the virus. Feline junctional adhesion molecule 1 (fJAM-1) was identified as the functional receptor for FCV [39]. This was the first protein receptor identified for the *Caliciviruses* as demonstrated by studies showing that transfection of the fJAM-1 gene into non-permissive cells transferred sensitivity to infection that was blocked by antibodies to fJAM-1. JAM-1 is a member of the immunoglobulin-like superfamily of proteins found on the surface of leukocytes and blood platelets and is thought to regulate the formation of tight junctions in epithelial and endothelial cells. Domain deletion and mutagenesis experiments have mapped the virus interactions to outermost D1 domain [40]. It is interesting to note that bacterially expressed fJAM-1, which is devoid of any glycan modifications, is able to inhibit virus binding to cells [40]. This would suggest that the sialic acid on fJAM-1 is not necessary for binding of the receptor to FCV.

The cryo-TEM structure of FCV complexed with fJAM-1 was determined to a resolution of ~18Å [41]. While the general structure agreed well with the atomic structure of SMSV, it is interesting that either alone or complexed with fJAM-1, the C/C dimers were less ordered than the A/B dimers. In particular there was apparent disorder around the flexible junction between the S and P domains of the capsid protein.

The structure of human JAM-1 (hJAM-1) has been determined to atomic resolution [42]. In the crystal cell, the two D1 Ig-like domains interact at ~90° angles akin to what is observed in antibody structures. The D2 domains interact with different copies of the hJAM-1 proteins in an anti-parallel fashion. For a number of reasons, including possible interactions with reoviruses, it was suggested that these D1 interactions might be dynamic in nature. The structure of human JAM-1 was altered to reflect the sequence of fJAM-1 and fitted into the electron density envelope of the FCV/fJAM-1 complex [41]. Unlike the interactions observed in the crystal structure of hJAM-1, the model suggests that two copies of fJAM-1 bind to the outermost portion of the P2 dimers in a crossed manner such that the D1 domain from one bound molecule interacts with the D2 domain of the other. The current model, however, places these two domains fairly close together in some regions. Again, this model places a single D1 domain at the viral surface rather than the D1 dimers observed in the crystal structure. fJAM-1 interacts with the known hypervariable antigenic loops of P2 and those residues in fJAM-1 involved in these contacts have been shown to be important for virus binding.

This work has been updated with very recent and exciting cryo-EM studies on the FCV/VP2 complex [43]. In these cryo-electron microscopy studies, they found that the minor capsid protein, VP2, forms a large portal-like structure at a unique three-fold axis of symmetry, following receptor binding. This complex is not observed in the absence of receptor and is formed of twelve copies of VP2, with their hydrophobic N termini pointing away from the virion surface. Further, they suggest that structural changes at the portal site opens a pore in the capsid shell. They hypothesize that this portal acts as a channel to deliver the viral genome through the endosomal membrane. While it has shown that VP2 is critical for the production of infectious virus [44], this is the first time a detailed mode of action has been proposed. It should be noted that the FCV cryo-EM structure has the “compressed” structure, although the P domains are clearly flexible with regard to the capsid surface. This suggests that, at least for FCV, that the compressed state might be conformation required for cell binding.

## 6. MNV P Domain Flexibility and Antibody Escape

**Figure 6:**
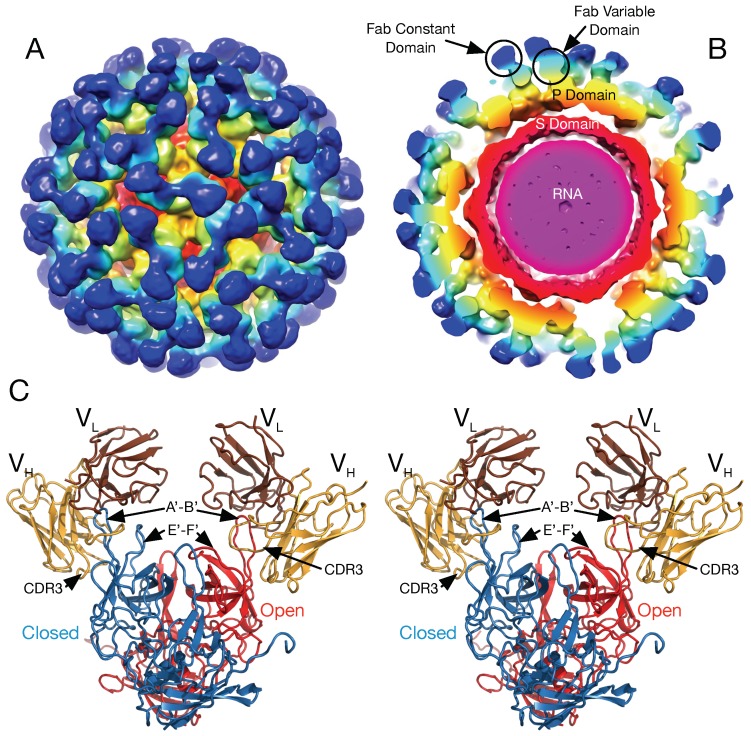
Pseudo atomic structure of MNV complexed with neutralizing antibody 2D3. Panel A shows the structure of MNV complexed with Fab fragments of 2D3 and panel B shows the same structure as a slice along the midsection. The surfaces are colored according to the distance from the center of the particle. As noted, the shell domain is orange, the P domain is yellow, the Fab variable domain is cyan, and the constant domain is blue. Note the large gap between the shell and P domain and that the Fab binds to the very top of the P domain. Panel C is a stereo diagram of the pseudo atomic model of the 2D3/MNV complex. The open and closed conformations of the P domain is shown in red and blue, respectively. The heavy chain variable and the light variable domains are shown in yellow and brown, respectively. Note that the CDR3 loop of the heavy chain fits into the cleft between the A’-B’ and E’-F’ loops in the open conformation but completely overlaps the E’-F’ loop in the closed conformation, making it unlikely that 2D3 can bind to the closed conformation. Figure adapted from [27].

**Figure 7:**
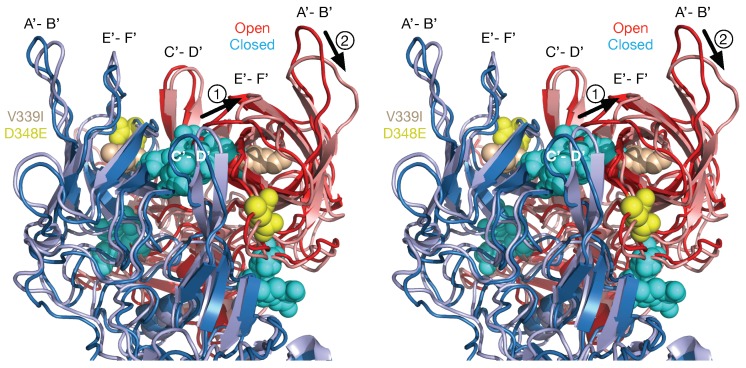
Dynamic simulations of the natural escape mutation to 2D3, V339I. Since V339I blocks 2D3 binding but is very distal to the antibody binding site, dynamic simulation calculations were performed to see the effects of V339I on the structure of the P domain dimer. The open conformation is red and the closed is blue with the darker hues representing the initial structure before the simulations. The location of V339I is shown in tan and the other escape mutant, D348E, in yellow. Compared to simulations on the wild type P domain structure, several notable changes were observed. There were disruptions in the hydrogen bonds between the dimers (cyan spheres) and the A’-B’ and E’-F’ loops in the open conformation started to approach that of the closed conformation. Figure adapted from [27].

**Figure 8:**
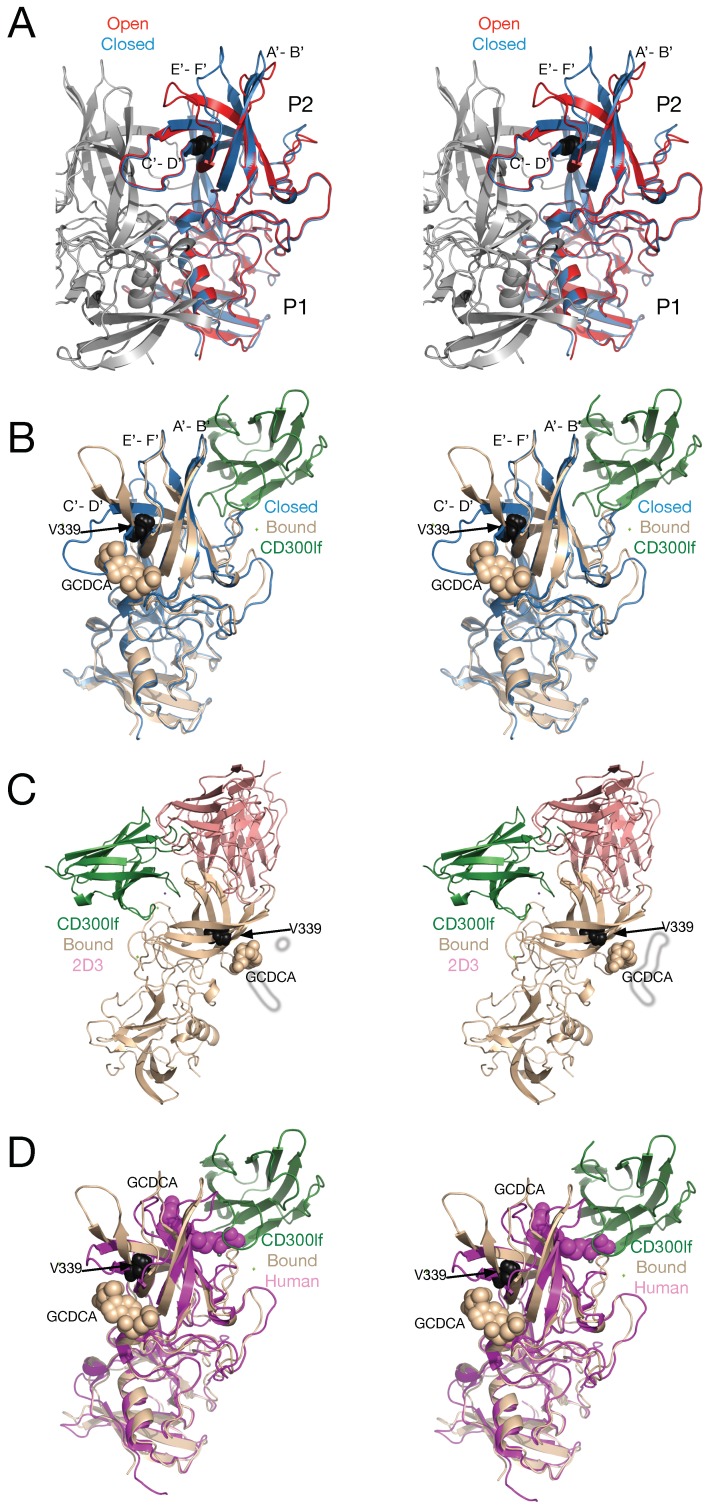
Comparisons of various P domain structures and complexes. Panel (**A**) shows an overlay of the open (red) and closed (blue) conformations observed in the MNV P domain crystal structure [19]. Panel (**B**) shows an overlay of the MNV P domain (tan ribbon) with receptor (green ribbon) and bile salts (tan spheres) onto the closed conformation (blue) from panel A. Also shown is the location of the V339I escape mutation to antibody 2D3 (black spheres). Note that the conformations of the A’-B’ and E’-F’ loops with the bound receptor is nearly identical to that of the closed conformation in panel (**A**). Also note that the V339I escape mutate is immediately adjacent to the bound bile salts. Panel (**C**) shows a hypothetical overlay of the P domain with 2D3 antibody (pink) and CD300lf receptor (green) bound. While their binding sites do not directly overlap, it is impossible for both to bind simultaneously. Panel (**D**) shows an overlay of the MNV receptor complex (tan) with the human P domain complexed with bile salts (mauve). Note that CD300lf in MNV directly overlaps the bound GCDCA in the human norovirus structure.

In the initial crystal structure of the MNV P domain [19], the outer two loops (A’–B’ and E’–F’) displayed two discreet conformations; a closed structure where the two loops are tightly associated and an open structure where the loops are splayed apart. This conformational difference is shown in Figure 6, Figure 7 and Figure 8. This “open” and “closed” designation will be used in the following discussion.

To understand how noroviruses escape antibody neutralization, we determined the cryo-EM structure of MNV complexed with the Fab fragment from a neutralizing A6.2 [14]. It was clear that A6.2 bound to the outermost tip of the P2 domain, right where the A’B’ and E’F’ loops lie. However, without the atomic structure of A6.2, it was not clear whether it was binding to open or closed conformations, or both. To better understand the interaction of mAb A6.2 with the P domain, the structure of the A6.2 Fab was determined to ~2.5 Å [45]. Typically, the third hypervariable loop (CDR3) of the heavy chain makes the majority of the contact with the epitope of the antigen [46]. Interestingly, this loop in A6.2 is strongly hydrophobic with the sequence “YFYALDYW”. When the structures of A6.2 and the P domain dimer was placed into the cryo-EM density and refined using COLLAGE in the SITUS package [47], it was evident that A6.2 fit better onto the open conformation than the closed, with the hydrophobic CDR3 loop extending into the hydrophobic interface between the A’-B’ and E’-F’ loops. Those hydrophobic residues in the P domain are deeply buried under the tips of the A’B’ and E’F’ loops in the closed conformation and not accessible to mAb A6.2. In addition, the CDR3 loop of A6.2 completely overlaps the E’-F’ loops in the closed conformation, making it impossible for A6.2 to bind. Taken together, the modeling strongly suggested that mAb A6.2 prefers the open conformation both in terms of structural and hydrophobic complementarity.

Using the A6.2/MNV-1 docking model [14,19] as a guide for mutagenesis, we identified six single point mutations in the E’F’ loop of the MNV-1 P domain that completely abrogated mAb A6.2 binding to MNV-1 and allowed mAb A6.2 neutralization escape in culture and in mice [45]. This structure, combined with the mutagenesis results, suggested that a number of escape mutants block antibody binding distal to the epitope by limiting the conformational repertoire of the mAb epitope in the E’F’ loop. These studies are the first to suggest that escape mutations may act by limiting flexibility of an epitope or by driving the conformation towards a weak binding structure. For example, in the open conformation, the L386F mutation would place the larger Phe side chain directly in contact with the bound antibody and would be exposed to water. However, in the closed conformation, the PHE would be completely buried between the A’-B’ and E’-F’ loops. Hence, the L386F mutation would appear to push the structural equilibrium towards the closed conformation—to which A6.2 cannot bind. A similar observation was made with A382R. The A382R mutation in the close conformation likely places the Arg into the solvent and thus should be the favorable structure. In contrast, in the open conformation, the A382R mutation would place the Arg side chain into a cluster of basic residues. Again, this suggests that the plastic P domain can thwart antibody binding by shifting the structural equilibrium away from the conformation favored by the neutralizing antibody.

An even more extreme case of P domain plasticity was observed with a second neutralizing antibody, 2D3 (Figure 6). Compared to A6.2, MNV1 had far more difficulty in overcoming neutralization to 2D3, as it took over 20 passages for MNV1 to escape mAb 2D3 neutralization [27,45]. The escape mutants to A6.2 were still neutralized by 2D3 and visa-versa. Importantly, all of the MNV strains tested were neutralized to at least some degree by 2D3 while A6.2 was far more selective. This suggested that the epitope on the P domain region recognized by 2D3 was significantly different and more conserved than that for A6.2 and would therefore make a good vaccine target.

To understand the difference between these two antibodies, the cryo-EM structure of the 2D3 bound to MNV was determined [27] (Figure 6). Like A6.2, 2D3 appears to bind to the open but not the closed conformation of the P domain. While mAb 2D3 contacts the E’-F’ loop of the P domain similar to mAb A6.2, mAb 2D3 appears to bind slightly deeper in the crevice between the A’-B’ and the E’-F’ loops. Surprisingly, neither of the natural escape mutants to 2D3 (D348E and V339I) are actually in contact with the bound antibody. It is highly unusual that none of the possible mutations at the paratope/epitope interface produced viable virus. Just as odd is the fact that even though some of the escape mutations to A6.2 are in contact with 2D3, none of them can escape 2D3 neutralization. What is so special about the 2D3 contact area that severely restricts the repertoire of possible mutations so that only “allosteric” escape mutants were isolated?

To address how the 2D3 escape mutants, that are distal to antibody contact and buried beneath the surface, can block 2D3 binding, dynamic simulations were performed on the P domain where the V339I mutation was modeled into both the open and closed conformations [48] (Figure 7). The results for the three simulations indicate that FabD/MNV bound complex is sensitive to the flexibility of P domain dimer interfacial interactions, in particular the salt bridge network that undergoes rearrangements necessary to accommodate the antibody binding. The buried dimer interface salt bridge network is complex and dynamic allowing the dimer to adopt more than one conformation. For the V339I mutation, a mutation in neither the A’–B’ or E’–F’ binding loops changes the salt bridge network and restricts the molecular liberation of the interfacial region and thus the conformational flexibility. Side chain interactions in folding differ from those found at interfaces where association accommodates minor conformational adjustments, usually in side chains, not in the folded state of the protein units. The salt bridge and hydrogen bonding network at these buried interfaces, void of mediating solvent molecules, are specific and complementary. Salt bridges are able to contribute to conformational specificity and contribute to molecular recognition and catalysis. Thus, as discussed by DeGrado et al. [49] salt bridges are difficult to predict because of the cost of formation, dehydration of a basic residue and carboxylate and the electrostatic and hydrogen bonding interactions, but they may be viable designable interactions.

Together, these results strongly support the contention that the P domain is in a dynamic structural equilibrium where the crystallographically observed open and closed states represent two possibilities of a montage of conformations. The site directed escape mutations to A6.2 and the naturally occurring escape mutations to 2D3 suggest that the virus can block antibody binding by shifting this equilibrium towards the closed state. If true, then it necessarily follows that the closed state is the viable and infectious form of the virus. Further, it is not at all clear why the 2D3 contacts on the P domain are so immutable and suggest important roles in this particular region of P domain such as receptor binding.

## 7. MNV/Receptor Interactions

The Virgin lab recently discovered that the cell receptor for MNV was proteinaceous and was not dependent upon carbohydrates for binding [50]. Using CRISPR-Cas9 technology, a gene that encodes a cell-surface protein that contained an immunoglobin domain and belonged to a lipid protein family was most significantly enriched in the surviving cells, *Cd300lf*. It was confirmed that this was the receptor from studies showing that knocking out Cd300lf in BV2 cells blocked infection by MNV. Further, treating the cells with antibodies to CD300lf blocked MNV attachment. Attachment of MNV to BV2 cells was not affected by pre-treatment of the cells with the mannosidase I inhibitor, kifunensine, suggesting that carbohydrates do not play a significant role in MNV attachment. Even stronger evidence of the importance of CD300lf came from the demonstration that expression of this mouse cell surface protein in HeLa cells made these human cells susceptible to MNV infection. While carbohydrates are not apparently important for cell binding, they did find that a serum cofactor was required for cell attachment. Recent studies on MNV demonstrated that these some of these serum factors are bile salts [26]. In these studies, they showed that the bile acid glycochenodeoxycholic acid (GCDCA) enhances viral attachment to BV2 cells. This is clearly a specific interaction since a chemically similar salt, taurocholic acid (TCA), had no effect on binding. These results were further substantiated using isothermal titration calorimetry that showed GCDCA binds to the expressed form of the P domain with Kd of ~6µM, but TCA did not bind at all.

Using these soluble forms of CD300lf and the P domains, they also directly measured the interaction affinity using surface plasmon resonance [26]. The monomeric CD300lf protein bound to the P domain with a Kd of ~219 µM. While this represents a very weak interaction, the other members of the CD300 family, that failed to confer susceptibility to MNV infection (mouse CD300ld, mouse CD300lh, and human CD300f), showed little to no binding. By adding calcium, the affinity improved to ~25 µM and when GCDCA and calcium were both added, the affinity improved to ~12 µM. The monomeric CD300lf molecules were then linked as dimers by fusing them to an antibody Fc fragment. As expected, this improved apparent affinity (avidity) to ~0.5 µM. This is entirely consistent with foundational studies demonstrating that Fab fragments bind with ~100–1000 lower apparent affinity than the corresponding IgG [51]. The neutralizing antibody, A6.2, was able to block binding of this bivalent CD300lf molecule, suggesting that at least in-vitro antibody neutralization is due to blocking of receptor attachment.

## 8. Overview of Structural Studies

Figure 8 shows a summary of some of the structural studies on norovirus P domain. In panel A, the open (red) and closed (blue) conformations of the MNV P domain monomers are compared. Shown in grey is the other copy of the P domain that forms the dimer. Note that the A’-B’ and E’-F’ loops are splayed apart in the open (red) conformation but tightly associated in the closed (blue) conformation. The structure of the tip of the P domain in solution is more than likely to exist in a structural equilibrium including at least these two conformations.

The details of the MNV/receptor interactions have been elucidated with the atomic structure of the P domain/CD300lf complex [26,52] (Figure 8B). The CD300lf contact site is near the top of the P2 domain, between the A’–B’ and D’–E’ loops. Interestingly, this contact surface includes some of the amino acids involved in the binding of the neutralizing antibody, A6.2. From structural analysis, they also found that that that GCDCA (tan spheres) and lithocholic acid (LCA) bind in two deep pockets in the P domain dimer interface, distant from receptor and antibody binding sites [26]. The location of the 2D3 escape mutation is highlighted by black spheres.

Interestingly, the conformation of the A’–B’ and E’–F’ loops in the receptor complex is nearly identical to that of the closed conformation. The major difference is in the C’–D’ loop that is “turned up” compared to the P domain alone. This affords enough space for the bile salt, GCDCA (tan spheres), to bind. It may be that the structural equilibrium in solution is shifted by GCDCA binding to the closed form, which may be preferred by CD300lf. In this way, the bile salts may enhance receptor binding, indirectly, by altering the dynamics and structural equilibrium of the P domain. What is particularly interesting is that one of the two escape mutants to the neutralizing antibody, 2D3, is the buried residue V339 (black spheres) that lies immediately adjacent to the bile salt binding site. As with the bile salts, we had proposed that the V339I mutation shifted the equilibrium towards the closed conformation that does not favor antibody binding [20,45,48]. It is rather exciting to consider that the V339I escape mutant and bile salts drive the P domain conformation towards the closed conformation that favors receptor binding and at the same time away from the open conformation favored by antibody binding.

Panel C shows the binding locations of CD300lf (green) and the neutralizing antibody 2D3 (pink) with respect to the bile salt binding site. This figure is rotated 90° compared to panels A and B and is hypothetical since the receptor and antibody do not bind at the same time. As this figure shows, the two proteins do not bind to the exactly the same location on the P domain but the main body of the proteins are too close together to bind simultaneously. It should be noted, however, that whether antibodies overlap the binding site of CD300lf in the case of MNV or HGBA’s in the case of human noroviruses, neither circumstances are likely to be necessary for antibody efficacy. As discussed previously [53,54], it must be remembered that the length of an intact antibody is approximately the radius of these particles and therefore binding is likely to greatly affect the closest approach of the virus to the cell surface regardless of the exact binding location on the virus. Further, in-vivo, binding alone is sufficient for recognition by the other immune system components. Therefore, the locations of antibody binding are likely to be more critical with regard to binding affinity and viral protein conservation than whether the paratopes overlap receptor binding. This figure also exemplifies how far the V339I escape mutant and GCDCA are to antibody and receptor binding sites. The only explanation for how this escape mutant works is that the P domain is a highly dynamic structure that can be affected by ligands and mutants in an allosteric manner.

Panel D shows a comparison between the MNV P domain/CD300lf/GCDCA complex [26] and the human norovirus GII.1 (mauve) complexed with GCDCA [28]. What is particularly interesting is that human GII.1 binds GCDCA (mauve spheres) at the very top of the P2 domain rather than at the dimer interface closer to the P1 domain. Importantly, GCDCA in GII.1 overlaps with the location of CD300lf in MNV and is immediately adjacent to the HGBA binding location in human norovirus GII. Indeed, these authors suggested that bile salts cause conformational changes in these upper P2 domain loops that allow the polysaccharides to bind [28] and allow some genotypes to bind HGBA’s that they were unable bind previously. Therefore, while the details are different, both GII.1 and MNV are clearly dynamic proteins in conformational equilibriums. External stimuli appear to be able to alter this equilibrium to favor receptor binding.

**Figure 9:**
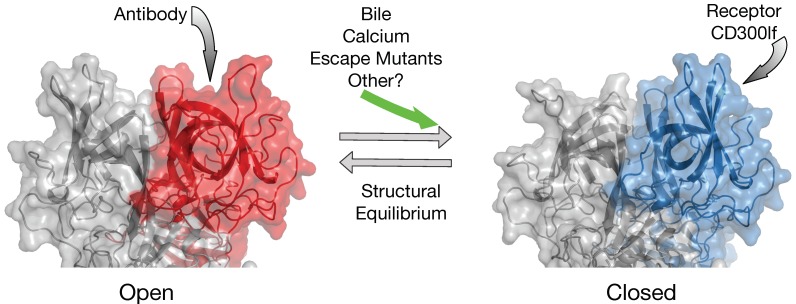
Model for intrinsic conformational dynamics within the P domain. In this highly simplistic overview, there are at least two conformation for the P domain; open (red) and closed (blue). The antibodies to date that neutralize MNV appear to only bind to the open conformation while the receptor appears to prefer binding to the closed conformation. From the results reviewed here, it appears that the structural equilibrium is shifted towards the receptor-binding closed state by some of the “allosteric” antibody escape mutations and by external stimuli such as bile salts and metals like calcium.

Figure 9 shows a possible model for the structural dynamics within the P domain. The conformation of the P domain in solution is more than likely a montage of structures including the observed open and closed configurations. This model suggests that the structural equilibrium can be disrupted by environmental cues and antibody escape mutants. In the open conformation, the A’-B’ and E’-F’ loops are splayed apart making room for antibodies (e.g., A6.2 and 2D3) to bind. The closed conformation does not expose the hydrophobic region between the two loops and the E’-F’ loop clashes with the CDR3 loop, therefore preventing antibody binding. All of the escape mutations to 2D3 are distal to the antibody binding contact and dynamic simulation studies with the V339I escape mutation suggest that it is causes long range structural disruption in the P domain dimer and may be pushing the structure from the open (that binds antibody) to the closed (that does not bind antibody) conformation [48]. Similar to the V339I escape mutant, compounds in the gut milieu such as calcium and bile salts may also push the structural equilibrium towards the closed conformation and it is apparently this conformation to which the receptor binds [26]. Interestingly, the location of V339I is immediately adjacent to the bile salt binding site in MNV and may be mimicking the effects of bile salts. While clearly this simplistic model needs to be extensively tested, it suggests that the P domain in solution is sampling many conformations waiting for the right environmental cues when in the best location of the animal for infection. It is fascinating that apparently the predominant structure presented to, or recognized by, the immune system is the open conformation while the conformation necessary for receptor binding is closed. This could be akin to the ‘camouflage’ model proposed to FMDV where the integrin binding RGD motif is on a highly mobile loop that presents many different conformations to the immune system [55,56].

## 9. Summary

These studies have shown that the Calicivirus capsid is not merely a “tin can” moving viral genome from cell to cell. Instead, it a highly dynamic virus that appears to have a highly mobile P domain that can move around the capsid surface, likely to facilitate binding to the target cell. This flexibility of the P domain might also be involved in avoiding the immune system by presenting antigenic sites exposed in one state but not the other. There is similarly a high degree of mobility within the P domain with it adopting a spectrum of conformations depending upon various environmental cues. This flexibility may be to help it target the best tissue for infection. It might even be used to confuse the immune system by presenting a wide array of structures. In essence, could the virus be presenting one “face” to the immune system while adopting a very different one for cell attachment depending upon the environment?
